# Annotation of a hypothetical protein coding gene PAS_chr2-2_0152 containing Lysine Methyl transferase SMYD domain from Komagataella phaffii GS115

**DOI:** 10.6026/97320630015542

**Published:** 2019-08-31

**Authors:** Ridip Kumar Gogoi, Ringhoilal Chorei, Himanshu Kishore Prasad

**Affiliations:** 1 Functional Genomics Laboratory, Department of Life Science and Bioinformatics, Assam University, Silchar, Assam-788011, India

**Keywords:** Hypothetical protein, SMYD, Komagataella phaffii, phylogenetic analysis, methyltransferase

## Abstract

The methylotrophic yeast Komagataella phaffii is an industrial workhorse yeast species that has been widely used in biotechnology industries for recombinant protein production. Genome sequencing of this yeast in 2009 have enabled scientists to assign and characterize functions to most of its proteins while few hypothetical proteins remain uncharacterized. Therefore, it is of interest to characterize the hypothetical protein coding gene PAS_chr2-2_0152 as SET containing the ZNF-MYND (SMYD) domain. They share a homology with other methylotrophic and non-methylotrophic yeast species together with known SMYD proteins of Homo sapiens, with conserved distinctive SMYD domain patterns. A homology model is developed using the crystal structure of human histone-lysine methyl transferase smyd3 as template. These data points to that the hypothetical protein is a potential histones and non-histone lysine methyl transferase regulating cell cycle, chromatin remodeling, DNA damage response, homologous recombination and transcription in Komagataella phaffii. Data also suggests the evolutionary syntenic conservation of DNA damage regulator (RFX) and lysine methyl transferase (SMYD) genes in some yeast lineages, pointing to a conserved role requiring further confirmation.

## Background

With the advancement of the genomic era, genome sequence of the nonconventional methylotrophic budding yeast, Komagataella phaffii GS115 also known as Pichia pastoris has been published, which made it easier for the biologists to explore this yeast as a model system in utilizing its available resources. Accessibility of simple and vigorous high cell density cultivation methods with easy genetic manipulations, extraordinary strong promoters with tight regulation, secretion capabilities, and excellent post-translational modification has further enhanced the utilization of this yeast a step forward [1]. Genome sequencing has enabled to identify several functional elements within the genome yet left many interesting elements to explore. Among these interesting elements, some were found to be uncharacterized hypothetical proteins (HP_s_) which encode for gene products with unknown function as a result of lack of in vivo experimental evidence [2]. Characterizations of HPs through various available known techniques indispose their roles in cellular biology along with new structures, functions and pathway knowledge. With known commercial successes, the advanced engineering of K. phaffii has enriched its secretory capacity and post-translational modification pathways [3]. Protein methylation accounts as one of the most important posttranslational modifications in cellular organisms.

Methyltransferases are the major class of proteins responsible for post-translational protein methylation which ranges from approximately 1-2% of genes in a variety of eukaryotic and prokaryotic organisms. Methyltransferases are categorized into three major structural protein families as seven-beta-strand, SETdomain, and SPOUT. In eukaryotic cells, mostly protein methylation takes place at arginine and lysine residues [4]. Themodel yeast Saccharomyces cerevisiae genome encodes over 20 protein methyltransferases, of which 12 proteins belong to the SETdomain and half of which can recognize non-histone substrates in post-translational protein modification. SET-domain subfamily of methyltransferase can catalyze the methylation of histone proteins by modifying lysine 4 of histone H3 and has a central role in transcriptional and efficient gene expression regulation [5,6]. In this study, we identify, characterize and annotate the functional characteristics of the K. phaffii GS115 gene PAS_chr2-2_0152 as SMYD domain containing protein methyltransferase using an in silico approaches.

## Methodology

### Identification of SET domain proteins in K. phaffii GS115 genome

The complete genome sequence of K. phaffii GS115 was retrieved from the NCBI database (Accession: PRJNA39439, ID: 39439) [1]. The Pfam SET domain hidden markov model (hmm) profile (Pfam accession PF00856.28) was obtained from the Pfam database [7]. Using HMMER 3.1 [8], HMM search was carried out in default parameters using Pfam SET domain hmm profile as query against K. phaffii GS115 genome. The resulted proteins were further queried in NCBI BLASTP with default parameters using NR and UniProtKB/Swiss-Prot database. Global blast alignment tool was used to determine the percent identity of the NCBI BLASTP hits [9]. For further confirmation, the proteins were subjected to InterPro scan [10] analysis to study the domain organization. Argot 2.5 webserver was utilized for functional annotation of the identified protein [11].

### Sequence conservation and phylogenetic analysis

In order to study the sequence conservation of the protein, BLASTP hits from UniProtKb/Swiss-Prot and NR databases were downloaded from the NCBI protein database. The protein sequences were aligned with the help of MUSCLE software [12] and a neighbor-joining (NJ) phylogenetic tree was constructed by MegaX with 1000 bootstrap replication value choosing substitution model Jones-Taylor-Thornton (JTT) with uniform rates and pairwise deletion for gaps treatment data set [13]. Gene order synteny was studied by using NCBI's Sequence Viewer.

### Physicochemical properties and subcellular localization

The physicochemical properties like isoelectric point (pI), molecular weight (M. Wt), amino acid composition, atomic composition , charge (positive or negative), extinction coefficient(EC), aliphatic index (AI), instability index (II), grand average of hydropathicity (GRAVY) and estimated half-life were determined by using the ExPASy ProtParam tool (http://web.expasy.org/protparam) [14]. Subcellular localization of the protein is predicted by using DeepLoc 1.0 [15].

### Secondary structure prediction and homology modelling of K.phaffii PAS_chr2-2_0152 protein

The ab initio secondary structure of the hypothetical protein was predicted with the aid of PSIPRED [16]. Phyre2 was used to predict the three-dimensional homology modelling in normal mode with default parameters [17]. The 3D model obtained was visualized and edited by using Chimera 1.11 [18]. The validation of the 3D model was checked with Verify3D [19].

## Results and discussion

**Figure 1:**
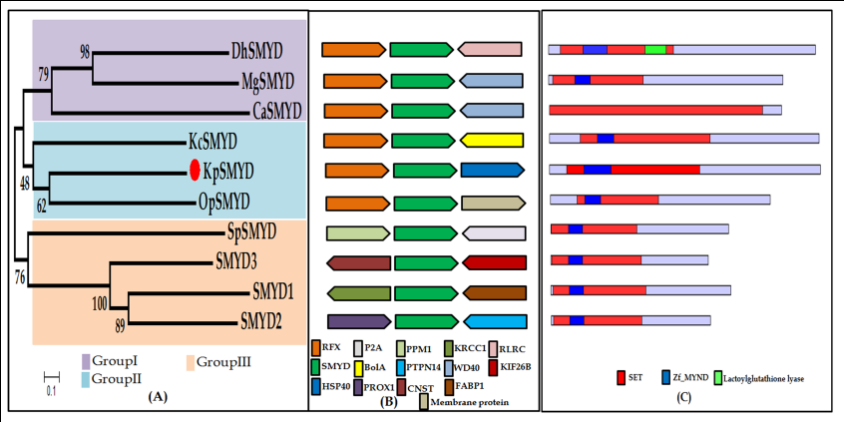
Phylogenetic analysis and sequence conservation of SMYD proteins (A). Neighbor Joining (NJ) phylogenetic tree representing KpSMYD protein with other SMYD proteins is shown. The accession numbers of the protein sequences were mentioned in Table 1. The tree was constructed from the multiple sequence alignment of whole SMYD protein sequences from BLASTP hits using NR and UniProtKB/Swiss-Prot database with 1000 bootstrap replicas following JTT substitution model with MegaX. (B) Study of the Synteny analysis of SMYD proteins using NCBI's Sequence Viewer. (C) SMYD domain organization in the SMYD proteins.

**Figure 2:**
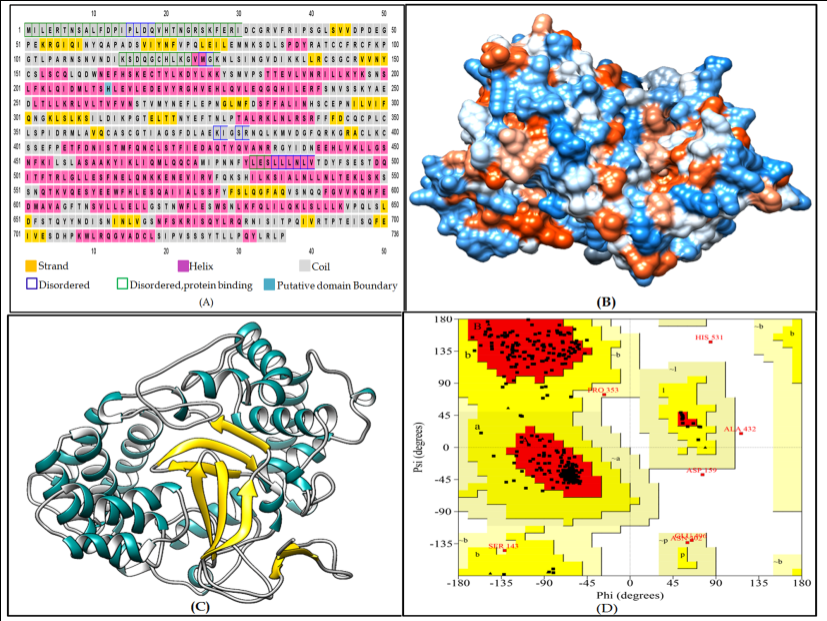
Structure analysis of the KpSMYD protein and cartoon representation of the 3D model (A) Secondary structure prediction using PSIPRED web server. (B) Hydrophobicity surface view of the 3D model predicted by Phyre2 using the template from PDB crystal structure molecule c3mekA chain A of Homo sapiens entitled human histone-lysine n-methyltransferase smyd3 in complex with sadenosyl- l-methionine. (C) Cartoon representation of the 3D model with the gray-colored coil, gold-colored strand, and dark cyan represents helix with white-colored inside. (D) Ramachandran Plot showing the validation of the modeled protein

The SET domain hmm profile search in K. phaffii GS115 genome identified eight SET domain-containing methyl transferase proteins. Domains analysis using InterProScan and Conserved Domain Database (CDD) tools further confirmed the presence of SET domains in the HMM search hits. BLASTP analysis using NCBI Non-redundant UniProtKB/SwissProt sequences databases identified known proteins in other organisms including S. cerevisiae. The protein with Gene Id: PAS_chr2-2_0152 (NCBI Accession No: XP_002492054.1, designated as hypothetical protein) was lacking any clear ortholog in the model S. cerevisiae genome. But the orthologous protein in other methylotrophic and nonmethylotrophic yeasts could be detected. BLASTP of XP_002492054.1 in UniProtKb/Swiss-Prot database identifies several SET and Smyd proteins in the Schizosaccharomyces pombe and Homo sapiens genomes. We named this hypothetical protein as KpSMYD as it harbors a unique SET (Suppressor of variegation, Enhancer of Zeste, Trithorax) and ZNF-MYND (Zinc Finger- Myeloid-Nervy-DEAF1) domain (SMYD) as revealed by BLASTP and InterproScan. Based on its annotation as a hypothetical protein, we have chosen KpSMYD in the present study for In silico characterization. KpSMYD protein showed 16 to 22 % identities with other Smyd proteins using global blast alignment tool (Table 1). Sequence analysis of the KpSMYD identified the unique N terminal SMYD domain characteristics wherein the SET domain is split by ZNF-MYND domain (Figure 1C). The SET domain in Smyd proteins functions as the catalytic domain while Zinc finger bearing MYND domain mediates protein-protein interactions [20]. To understand the evolutionary relationship between yeast and human Smyd proteins a neighbor-joining (NJ) phylogenetic tree was created using aligned protein sequences. The NJ tree clustered Smyd proteins into three major groups. Group I comprised of yeast hypothetical proteins from D. hansenii (DhSMYD), M. guilliermondii (MgSMYD) and C. albicans (CaSMYD). The group II consisted of methylotrophic hypothetical proteins belonging to K. phaffii (KpSMYD), K. capsulata (KcSMYD) and O. parapolymorpha (OpSMYD). In group III, Homo sapiens SMYD1p, SMYD2p, SMYD3p, and S. pombe SET6p formed a clade (Figure 1A). The physicochemical property of the KpSMYD protein was predicted by the ExPASy ProtParam server. The molecular weight of the protein is predicted to be about 84057.66 Da and reported to be a stable protein with instability index of 39.19.The protein is found to be hydrophilic as the predicted theoretical isoelectric point (pI) is 6.89 and a negative gravy value (-0.142) prompts the protein to be soluble. Higher aliphatic index of 96.78 indicates the protein to be stable over a wide range of temperature. The nucleus is the predominant localization of this protein as predicted by DeepLoc1 server. Synteny blocks are defined as the chromosomal regions of different genomes which share a common order of homologous genes [21]. Moreover, genes involved in several primary and secondary metabolisms are known to be clustered in the genomes of several fungi, S. cerevisiae, and other yeast species. The gene order analysis in the NCBI's sequence viewer identified the 5'neighbouring gene of the PAS_chr2-2_0152 (coding KpSMYD) as an annotated gene coding for a major transcriptional repressor of DNA-damage-regulated genes (PAS_chr2-2_0153, XP_002492053.1, Regulatory factor X, RFX1). The syntenic analysis identified the cooccurrenc of orthologous genes for RFX1 and KpSMYD to be conserved in other yeasts also. However, this conservation was not observed in the yeast S.pombe and Homo sapiens genomes (Figure 1B). Remarkable, RFX1 and its orthologs are conserved in yeasts, nematode and, vertebrates. In yeast, it is involved in the DNA damage and replication checkpoint pathway and acts as a major transcriptional repressor of DNA-damage-regulated genes. In contrast, the 3' neighboring gene order is not found to be conserved. DNaJ, Type II HSP40 co-chaperone is the next gene localized near to the 3' of the KpSMYD gene on chromosome number 2. The gene structure of KpSMDY is found to be represented by two exon counts, SpSMYD by three exon count and KcSMYD, OpSMYD, DhSMYD, MgSMYD, and CaSMYD by one exon count. The Homo sapiens SMYD1, SMYD2, SMYD3 is found to have exon count as 10, 12 and 30 respectively. In order to assign function to the identified hypothetical protein-coding gene of the K. phaffii, the protein sequence was uploaded in the ARGOT2.5 server by the selection of FunTaxIS then GO Consortium, HMMer models option with a total score (≥ 0): 50 and smiGIC as Semantic similarity metrics, which predicts the molecular function of the protein to be associated with metal ion binding (GO:0046872) and transferase activity (GO:0016740). Methylation (GO:0032259), histone methylation (GO:0034968), histone modification (GO:0016570), skeletal muscle organ development (GO:0060538), heart morphogenesis (GO:0003007) and heart development (GO:0007507) were the predicted biological processes. The predicted cellular component is nucleus (GO:0005634), cytosol (GO:0005829) and cytoplasm (GO:0005737). Secondary structure prediction helps in understanding the overall structural categories of proteins. It also helps in determining how the protein folds and give insights into its functional annotation [22]. PSIPRED predicted the secondary structure of the protein to be composed of 9.64% β-strand, 43.6% of α helices and 46.7% to be coiled-coil elements (Figure 2A). Modelling of the KpSMYD structure was accomplished using Protein Homology/analogY Recognition Engine V 2.0 (Phyre2) using normal mode [23]. The N-terminal region of the hypothetical protein KpSMYD was modelled with 100% confidence (Table 2). The Phyre2 server generated a top model with 19% identity with 100.0% confidence by the single highest scoring template c3mekA. The N terminal SMYD domain from position 39 to 542 (408 residues, 55% of the total) of the KpSMYD protein sequence was modelled using the above template. The template is a human histone-lysine n-methyltransferase smyd3 in complex with sadenosyl- l-methionine. Our analysis thus points to the conservation of the human SMYD domain in the hypothetical protein KpSMYD. The disordered region of the modelled protein is 14% suggesting regions with a diverged role as they are dynamically flexible. The modelled structure was refined with the help of MODrefiner [26] (Figure 2B, 2C)) and was validated by Verify3D, which shows that 91.3% of the residues of the modelled domain was in the favored region of Ramachandran plot (Figure 2D). The SYMD domain containing proteins mediate their action by methylating lysine amino acid residues of histones and non-histone targets thus controlling cellular processes like transcriptional activation, and repression, chromatin remodeling, cell cycle control, signal transduction pathways, pathology and physiology of skeletal and cardiac muscle [20,25]. Recently, human Smyd3 protein is shown to promote homologous recombination via regulation of H3K4- mediated Gene Expression [24]. Since the hypothetical protein KpSMYD of K. phaffii GS115 shares the 3D fold of the regulatory SMYD domain-containing proteins, they might share the likely cellular functions. Furthermore, the conservation of RFX and SMYD gene pairs in related yeast species points to a potential role in the pathway of DNA damage regulation.

## Conclusion

Genome sequencing of an organism involves gene annotation and curation of genomic sequences from gene to protein functions. We describe the annotation of hypothetical protein PAS_chr2-2_0152 from K. phaffii GS115 genome and linked it to a SMYD domain containing protein. The human SMYD domain containing proteins are known to be lysine methyl transferase enzyme family in the regulation of cell cycle and differentiation. SMYD proteins are also involved in other cellular functions like transcriptional activation and repression, protein-protein interactions and DNA damage response. The hypothetical protein is orthologous with the known yeast model S. pombe and also with other methylotrophic yeasts sharing similar synteny. A homology model of the hypothetical protein was developed using the human SMYD3 protein as the template while SMYD3 promotes homologous recombination via regulation of H3K4-mediated gene expression. Interestingly, the synteny analysis suggests the DNA damage regulator RFX and KpSMYD proteins are conserved in various yeast lineages, suggesting similar function. Thus, the potential function of the hypothetical protein is homologous recombination and/or associated with DNA damage response. These observations need further functional validation.

## Figures and Tables

**Table 1:** Blast global alignment of PAS_chr2-2_0152 (KpSMYD protein) (Accession No. XP_002492054.1) with Hits from BLASTP using NR and UniProtKb/Swiss-Prot database

S. No	Name Assigned	Accession No.	Protein Description	Organism	Length (AA)	Percent Identity (%)
1	MgSMYD	XP_001482836.1	Hypothetical protein	Meyerozyma guilliermondii ATCC 6260	637	20
2	OpSMYD	XP_013935809.1	Hypothetical protein	Ogataea parapolymorpha DL-1	597	22
3	KcSMYD	XP_022459850.1	Uncharacterized protein	Kuraishia capsulata CBS 1993	732	22
4	DhSMYD	XP_462014.2	DEHA2G10846p	Debaryomyces hansenii CBS767	725	21
5	CaSMYD	XP_713490.2	Hypothetical protein	Candida albicans SC5314	630	17
6	SpSMYD	NP_596514.1	putative histone lysine methyltransferase Set6	Schizosaccharomyces pombe	483	16
7	SMYD1	NP_001317293.1	SMYD1 isoform 1	Homo sapiens	490	17
8	SMYD2	NP_064582.2	SMYD2	Homo sapiens	433	16
9	SMYD3	NP_001161212.1	SMYD3 isoform1	Homo sapiens	428	17

**Table 2:** Data for a homology model of KpSMYD (PAS_chr2-2_0152) using Phyre2 is given. Secondary structure information of the modeled protein is presented in the table with the template information.

Protein Model	Residues		Resolution (Å)	Template Information				Sequence identity to the template (%)	Organism	Secondary structure Information		
	modeled at 100% confidence											
	Residues	Coverage (%)		PDB	Chain	PDB Header	Description			Disordered	Alpha helix (%)	Beta strand (%)
										(%)		
PAS_chr2-2_0152	408	55	1.75	3MEK	A	Trans	SET and ZNF-MYND domain containing protein 3	19	Homo sapiens	14	53	8
						ferase
